# Causal associations and genetic overlap between COVID-19 and intelligence

**DOI:** 10.1093/qjmed/hcad122

**Published:** 2023-06-07

**Authors:** Hongbao Cao, Ancha Baranova, Yuqing Song, Jian-Huan Chen, Fuquan Zhang

**Affiliations:** School of Systems Biology, George Mason University, Manassas, VA 20110, USA; School of Systems Biology, George Mason University, Manassas, VA 20110, USA; Research Centre for Medical Genetics, Moscow 115478, Russia; Institute of Mental Health, Peking University Sixth Hospital; NHC Key Laboratory of Mental Health (Peking University), National Clinical Research Center for Mental Disorders (Peking University Sixth Hospital), Beijing 100191, China; Laboratory of Genomic and Precision Medicine, Wuxi School of Medicine, Jiangnan University, Wuxi 214122, China; Institute of Neuropsychiatry, The Affiliated Brain Hospital of Nanjing Medical University, Nanjing 210029, China; Department of Psychiatry, The Affiliated Brain Hospital of Nanjing Medical University, Nanjing 210029, China

## Abstract

**Objective:**

COVID-19 might cause neuroinflammation in the brain, which could decrease neurocognitive function. We aimed to evaluate the causal associations and genetic overlap between COVID-19 and intelligence.

**Methods:**

We performed Mendelian randomization (MR) analyses to assess potential associations between three COVID-19 outcomes and intelligence (*N* = 269 867). The COVID phenotypes included severe acute respiratory syndrome coronavirus 2 (SARS-CoV-2) infection (*N* = 2 501 486), hospitalized COVID-19 (*N* = 1 965 329) and critical COVID-19 (*N* = 743 167). Genome-wide risk genes were compared between the genome-wide association study (GWAS) datasets on hospitalized COVID-19 and intelligence. In addition, functional pathways were constructed to explore molecular connections between COVID-19 and intelligence.

**Results:**

The MR analyses indicated that genetic liabilities to SARS-CoV-2 infection (odds ratio [OR]: 0.965, 95% confidence interval [CI]: 0.939–0.993) and critical COVID-19 (OR: 0.989, 95% CI: 0.979–0.999) confer causal effects on intelligence. There was suggestive evidence supporting the causal effect of hospitalized COVID-19 on intelligence (OR: 0.988, 95% CI: 0.972–1.003). Hospitalized COVID-19 and intelligence share 10 risk genes within 2 genomic loci, including *MAPT* and *WNT3*. Enrichment analysis showed that these genes are functionally connected within distinct subnetworks of 30 phenotypes linked to cognitive decline. The functional pathway revealed that COVID-19-driven pathological changes within the brain and multiple peripheral systems may lead to cognitive impairment.

**Conclusions:**

Our study suggests that COVID-19 may exert a detrimental effect on intelligence. The tau protein and Wnt signaling may mediate the influence of COVID-19 on intelligence.

## Introduction

Infection with severe acute respiratory syndrome coronavirus 2 (SARS-CoV-2), known as COVID-19, has an average infection fatality rate of approximately 0.5–2% in most locations worldwide.^1^ A number of risk or protective factors for COVID-19 outcomes have been reported, including neuropsychiatric diseases.^2–12^ Meanwhile, a sizable subpopulation of individuals who recovered from acute COVID-19 may suffer from a variety of lingering symptoms, collectively known as long COVID-19.^13–17^

SARS-CoV-2 can infect cells within the lower respiratory tract (trachea and lungs) and the upper respiratory tract (sinuses, nose and throat),^18^ in addition to damaging a wide range of human organs and systems, such as the immune system,^19^ nervous system^20^ and microvessels.^21^ Neuropsychiatric manifestations are common among individuals with COVID-19.^22^ Moreover, it has also been shown that COVID-19 could lead to a loss of 0.2–2% of brain tissue in regions processing the sense of smell and taste, as well as supporting higher functions; these losses are typically more pronounced among older individuals.^23^ A longitudinal magnetic resonance imaging (MRI) study revealed that individuals who contracted COVID-19 infection, on average, show more pronounced age-associated reductions in brain size and gray matter thickness as well as a larger cognitive decline than controls.^24^ It was reported that recovered COVID-19 patients have a higher risk of memory decline.^25^ The neurological sequelae of COVID-19 are associated with increased mental stress and the risks for mental disorders.^14^^,^^26–29^

It is worth mentioning that many of the peripheral pathological changes observed in COVID-19 patients are directly or indirectly linked to cognition.^30^ Recently, several studies have tested the relationship between COVID-19 and intelligence. In particular, Li et al.^8^ showed that education may act independently and jointly with intelligence in improving COVID-19 outcomes. Zhu *et al.*^10^ suggested a causal genetic linkage between an increased risk of symptomatic COVID-19 and decreased intelligence in children. A significantly increased risk of newly diagnosed Alzheimer’s disease was noted within 360 days after the initial COVID-19 diagnosis in elderly people.^31^ All of the evidence prompts a detailed evaluation of the relationships between COVID-19 and general intelligence.

Here, we hypothesize that COVID-19 may exert a detrimental effect on intelligence. We sought to evaluate the effects by using the Mendelian randomization (MR) framework applied to genome-wide association study (GWAS) summary results. Using large-scale automated mining of the literature, we also constructed functional pathways connecting COVID-19 and cognitive function.

## Methods

### GWAS summary datasets

The study utilized publicly available GWAS summary results, with all the participants of European origin. The summary results of the GWAS for intelligence contained 269 867 participants, including those from the UK Biobank (UKB).^32^ The COVID-19 datasets from the European population were obtained from the COVID-19 HGI GWAS round 7 (release date: 8 April 2022, without the 23andMe cohort).^33^ To avoid sample overlaps in the MR analysis, we selected the COVID-19 datasets without UKB participants, including hospitalized COVID-19 (40 929 hospitalized cases and 1 924 400 controls), critical COVID-19 (very severe respiratory confirmed 17 472 cases and 725 695 controls) and SARS-CoV-2 infection (143 839 virus-positive cases and 2 357 647 controls). In the identification of overlapping genomic loci between COVID-19 and intelligence, the hospitalized COVID-19 dataset of the European population, including 32 519 hospitalized cases and 2 062 805 controls, was utilized. The latter dataset included the UKB population. The SARS-CoV-2 infection dataset mainly reflects the susceptibility to the virus. The hospitalized COVID-19 and critical COVID-19 datasets characterize the severity of the disease, which we collectively called ‘severe COVID-19’ in this study. Ethical approval had been obtained from each of the original studies.

### MR analysis

The analyses were conducted using three complementary methods from TwoSampleMR,^34^ including weighted median (WM), inverse variance weighted (IVW) and MR-Egger. These models have different assumptions on pleiotropy.^35^ The IVW model was used as the primary MR method, which assumes an intercept of zero and estimates the causality by a fixed-effect model.^36^ The WM and MR-Egger models are more sensitive to horizontal pleiotropy but less powerful than IVW. The intercept of the MR-Egger regression was employed to assess the average horizontal pleiotropy.^35^

For each exposure phenotype, genome-wide significant single-nucleotide polymorphisms (SNPs) (*P* < 5 × 10^−8^) were selected as candidate instrumental variables (IVs). Then, these candidate IVs were pruned by a clumping *r*^2^ value of 0.001 within a 10-Mb window. The 1000 Genomes Project Phase 3 (EUR) was used as the reference panel.

### Shared genomic loci between COVID-19 and intelligence

To identify genetic overlaps between COVID-19 and intelligence, we compared their respective GWAS datasets. For each dataset, we used Functional Mapping and Annotation software to identify LD-independent genomic loci and map SNPs to genes.^37^ Independent significant SNPs (IndSigSNPs) were identified by their *P* values (*P *≤* *5.0*E*−08) and their independence from each other (*r*^2^ < 0.6). The IndSigSNPs that were in LD with each other within a 500-kb window (*r*^2^ < 0.1) were called lead SNPs. For each locus, regional associations were plotted by LocsZoom.^38^

### Protein–protein interaction analysis and pathway construction

The protein-coding genes shared between the sets identified for hospitalized COVID-19 and intelligence were used for the protein–protein interaction (PPI) analysis using STRING v11,^39^ followed by a subnetwork enrichment analysis (SNEA).^40^

To explore the molecular network alterations caused by COVID-19 and their influences on intelligence, we constructed functional pathways connecting these two entities using large-scale mining of the literature with Pathway Studio (www.pathwaystudio.com). The following criteria were applied to select the COVID-19-driven cognition/intelligence regulators: (i) the direction of the effect was from COVID-19 to cognition; (ii) exerted changes were in brain regions and other tissues linked to cognition/intelligence; and (iii) the supporting references passed quality control through manual inspection. The relationships that survived the filtering were used to construct the COVID-19-driven signaling pathways that may influence intelligence.

## Results

### MR analysis

**Figure 1.**
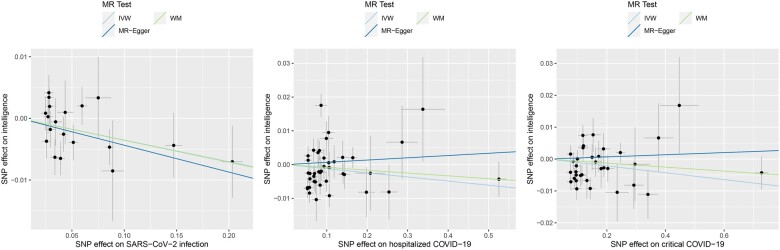
Causal effects of the COVID-19 outcomes on intelligence. The trait on the *x*-axis denotes exposure, the trait on the *y*-axis denotes outcome and each cross point represents an instrumental variant. The lines denote the effect sizes (*b*) of an exposure on an outcome.

In the MR analysis of the causal effects of the three COVID-19 phenotypes on intelligence, a total of 19, 41 and 34 IVs were extracted for SARS-CoV-2 infection, hospitalized COVID-19 and critical COVID-19, respectively. We found that genetic liabilities to SARS-CoV-2 infection (odds ratio [OR]: 0.965, 95% confidence interval [CI]: 0.939–0.993, *P* = 0.015) and critical COVID-19 (OR: 0.989, 95% CI: 0.979–0.999, *P* = 0.036) conferred causal effects on intelligence. There was suggestive evidence supporting the causal effect of hospitalized COVID-19 on intelligence (OR: 0.988, 95% CI: 0.972–1.003, *P* = 0.127) (Table 1 and Figure 1).

**Table 1.** Causal effects of the COVID-19 outcomes on intelligence

Exposure	Method	*b* (se)	OR [95%CI]	N_IV	Q_P	I^2^	Egger_intercept	P_pleiotropy	*P*
SARS-CoV-2 infection	IVW	-0.035 (0.014)	0.965 [0.939-0.993]	19	0.368	0.072	NA	NA	0.015
SARS-CoV-2 infection	WM	-0.036 (0.019)	0.965 [0.929-1.002]	19	NA	NA	NA	NA	0.066
SARS-CoV-2 infection	MR Egger	-0.044 (0.026)	0.957 [0.910-1.007]	19	0.316	0.063	0.001	0.697	0.109
Hospitalized COVID-19	IVW	-0.012 (0.008)	0.988 [0.972-1.003]	41	2.26E-06	0.579	NA	NA	0.127
Hospitalized COVID-19	WM	-0.008 (0.009)	0.992 [0.975-1.009]	41	NA	NA	NA	NA	0.343
Hospitalized COVID-19	MR Egger	0.007 (0.014)	1.007 [0.979-1.035]	41	8.07E-06	0.552	-0.002	0.123	0.644
Critical COVID-19	IVW	-0.011 (0.005)	0.989 [0.979-0.999]	34	7.36E-03	0.411	NA	NA	0.036
Critical COVID-19	WM	-0.006 (0.006)	0.994 [0.982-1.006]	34	NA	NA	NA	NA	0.306
Critical COVID-19	MR Egger	0.003 (0.009)	1.003 [0.986-1.021]	34	0.022	0.342	-0.003	0.061	0.702

IVW: inverse variance weighted; WM: weighted median; N_IV: number of instrumental variables; Q_P: Cochran’s *P* value of heterogeneity analysis.

The sensitivity analyses revealed that the directions of causal effect estimates across the methods were largely the same (Table 1 and Figure 1). Notably, tests of MR-Egger regression did not support directional pleiotropy in this MR analysis (MR-Egger intercept < 0.01, *P* > 0.05). Cochran’s test suggested possible heterogeneity in the hospitalized COVID-19 dataset and the critical COVID-19 dataset.

### Shared genomic loci influencing both COVID-19 and intelligence

**Figure 2.**
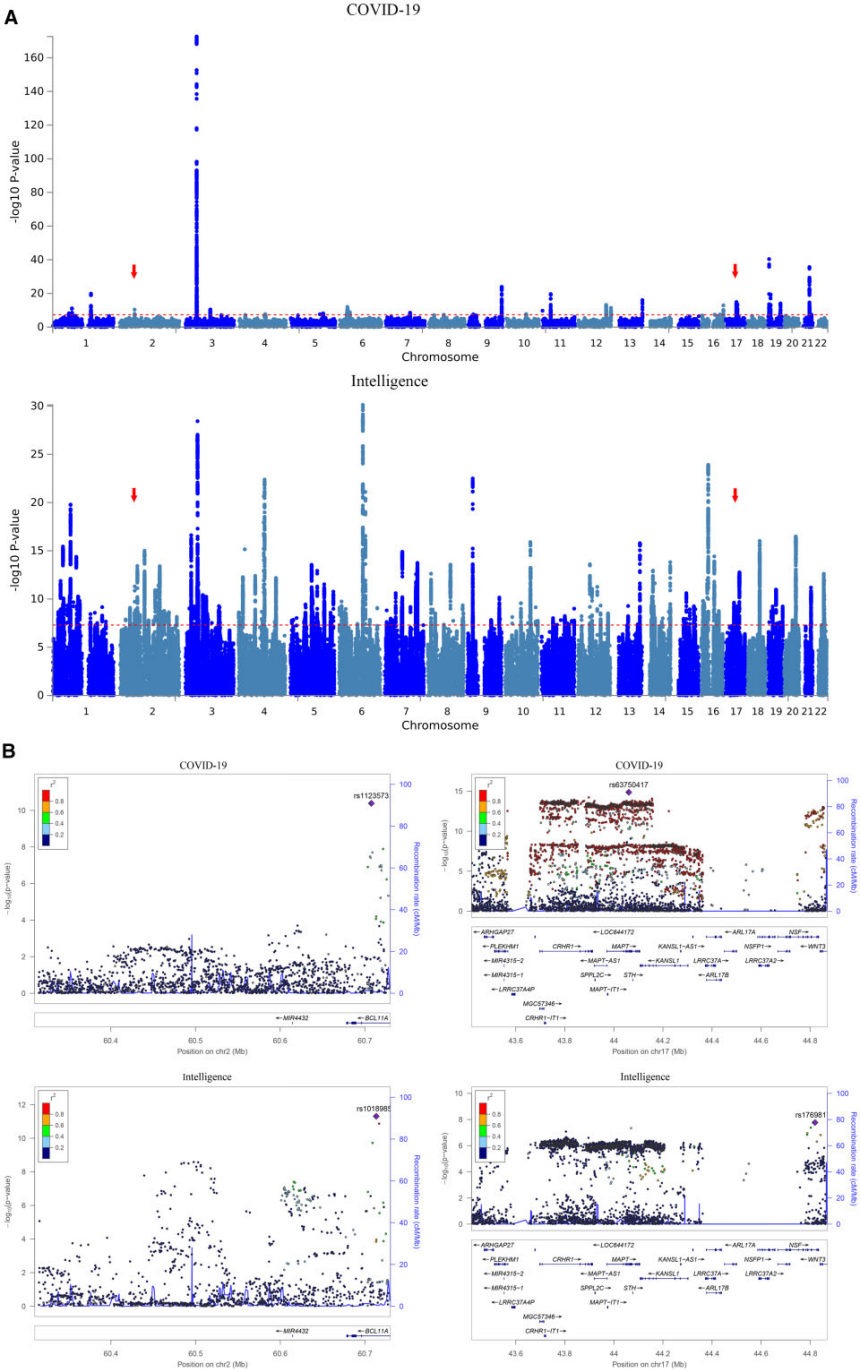
Overlapping genes between hospitalized COVID-19 and intelligence. (**A**) Manhattan plot of GWAS results of hospitalized COVID-19 and intelligence. The *x*-axis is the chromosomal position of SNPs and the *y*-axis is the significance of the SNPs (−log_10_P). Each horizontal dashed line denotes the genome-wide significance level of 5E-8. Red arrows indicate the two overlapping genomic loci between COVID-19 hospitalization and intelligence. (**B**) Two overlapping loci between hospitalized COVID-19 and intelligence. Left is the 2p16.1 locus and right is the 17q21.31 locus in hg19.

A total of 32 and 203 genomic loci were associated with COVID-19 and intelligence, respectively (Figure 2A and Supplementary Tables S1 and S2). Specifically, we detected two loci overlapping between COVID-19 and intelligence gene sets, including the 2p16.1 locus and the 17q21.31 locus (Table 2 and Figure 2). Ten genes overlapped between COVID-19 and intelligence gene sets included *BCL11A*, *MAPT*, *KANSL1*, *ARL17B*, *NSF*, *WNT3*, *LRRC37A*, *NSFP1*, *ARL17A* and *LRRC37A2*.

**Table 2.** Overlapping genomic loci between hospitalized COVID-19 and intelligence

Trait	SNP	CHR	BP	Start**:** End	A1/A2	*P*	Genes
Hospitalized COVID-19	rs1123573	2	60707588	60705232:60727416	G/A	4.13*E*−11	CL11A
Intelligence	rs10189857	2	60713235	60317457:60726427	A/G	4.91*E*−12	BCL11A
Hospitalized COVID-19	rs63750417	17	44060775	43422855:44865603	T/C	1.37*E*−15	ARHGAP27; PLEKHM1; DND1P1; RPS26P8; CRHR1-IT1; CRHR1; MAPT-AS1; SPPL2C; MAPT; STH; KANSL1; KANSL1-AS1; ARL17B; LRRC37A; NSFP1; ARL17A; LRRC37A2; NSF; RPS7P11; WNT3
Intelligence	rs17698176	17	44819595	44040184:44848314	T/G	1.70*E*−08	MAPT; KANSL1; ARL17B; NSF; WNT3; LRRC37A; NSFP1; ARL17A; LRRC37A2; FAM215B

CHR, chromosome; BP, base pair.

### PPI analysis and SNEA results

**Figure 3.**
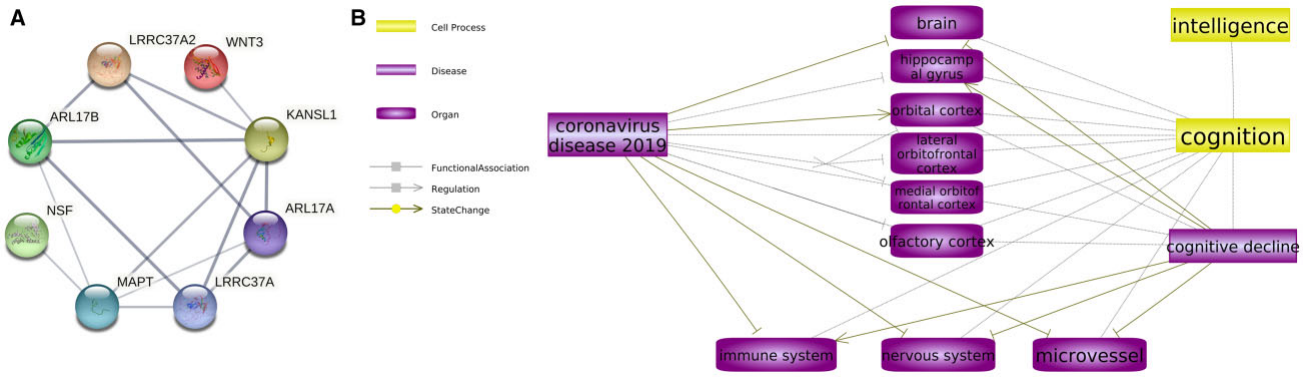
PPIs and COVID-19–intelligence connections. (**A**) PPIs between the shared protein-coding genes. (**B**) Biological abnormalities induced by COVID-19 at the organ and system levels contribute to the development of intelligence decline. The relation type ‘–|’ represents an inhibition and ‘→’ represents a relationship with no polarity.

Among the 10 overlapping genes, all except *NSFP1* were protein coding. PPI analysis showed that a majority of protein-coding genes formed an interconnected group, with *BCL11A* remaining an extant entity (Figure 3A).

The SNEA results showed that 6 out of these 10 genes were enriched within 37 disease-centered subnetworks (*P* < 0.05, Supplementary Figure S1 and Supplementary Table S3). Interestingly, 30 out of these 37 pathophysiological subnetworks were related to cognitive decline, indicating that these genes may contribute to the impairment of intelligence in a variety of contexts.

### Functional pathways connecting COVID-19 and intelligence decline

The analysis of data obtained from structural and functional MRI studies (Supplementary Table S4) allowed the construction of functional pathways that connect COVID-19 with changes in different brain regions. Figure 3B illustrates various noticeable alterations in brain structure resulting from COVID-19, such as decreased gray matter thickness and tissue contrast in the orbitofrontal cortex and parahippocampal gyrus, tissue damage in regions connected to the primary olfactory cortex and a reduction in overall brain size. These brain abnormalities often coincide with the pattern of cognitive decline associated with aging. Some of these changes may be attributed to COVID-19-induced dysfunction of the microvessels, while others could be caused by direct damage to the neuroglial and immune systems. Both of these pathophysiological processes have been linked to impaired cognition. The pathway depicted in Figure 3B provides a potential framework for understanding the possible connection between COVID-19 and cognitive decline at the level of observable traits.

## Discussion

In this study, we conducted an MR analysis to explore the potential causality between three forms of COVID-19 and intelligence. Our results showed the causal effects of SARS-CoV-2 infection and critical COVID-19 on intelligence, as well as the possible influence of hospitalized COVID-19 on intelligence, indicating that COVID-19 patients might be at risk of intelligence decline.

Our study shows that the genes located at the 2p16.1 and 17q21.31 regions influence both severe COVID-19 and intelligence. The 2p16.1 locus harbors the single protein-coding gene *BCL11A*, which plays a vital role in B and T lymphopoiesis^41^ and defines dendritic cell fate.^42^ Genetic variation within *BCL11A* determines residual levels of fetal hemoglobin,^43^ which may be protective against the symptoms of coronavirus infection.^44^ In undifferentiated epithelial cells, the product of this gene prevents senescence by accelerating the repair of oxidized DNA.^45^ During postnatal corticogenesis, *BCL11A* prevents the death of projection neurons.^46^ Haploinsufficiency of *BCL11A* underlines intellectual disability syndrome (IDS) associated with the hereditary persistence of fetal hemoglobin (HbF), also known as Dias-Logan syndrome^47^ and a chromosome 2p16.1p15 microdeletion syndrome.^48^ Peculiarly, *BCL11A* was previously reported as a genome-wide risk gene for COVID-19^49^ and as a pleiotropic gene for attention-deficit/hyperactivity disorder, autism spectrum disorder and intelligence.^50^^,^^51^ Notably, these three neurodevelopmental features are underpinned by shared genetics.^51^^,^^52^

The 17q21.31 locus contains eight overlapping protein-coding genes, including *MAPT*, *KANSL1*, *ARL17B*, *NSF*, *WNT3*, *LRRC37A*, *ARL17A* and *LRRC37A2.* PPI analysis showed that the respective proteins form an interconnected network (Figure 3A), which is functionally linked to a set of diseases associated with cognitive decline (SNEA results). The genes located within the contiguous region were repeatedly identified as contributors to COVID-19 phenotypes. For example, the chromatin modifier gene *KANSL1*, which is also a risk gene for atrial fibrillation and flutter as well as for pulmonary fibrosis, was identified in studies of genetic associations with severe COVID-19.^53^^,^^54^ The same gene serves as a pathogenic culprit for Koolen De Vries syndrome characterized by intellectual disability accompanied by characteristic facial features and hypotonia,^55^ a longevity gene^56^ and a contributor to Alzheimer’s disease phenotypes.^57^

The *MAPT* gene encodes the microtubule-associated protein tau. This gene was identified by our previous multi-omics integrative analyses as a contributor to COVID-19.^58^ A recent study reported that increased levels of tau in the blood, which is possibly due to its excretion by exosomes,^55^ are associated with fatal outcomes of COVID-19.^59^ Notably, by adhering to the SARS-CoV-2 S1 receptor-binding domain, tau protein precipitates the aggregation of amyloid-like proteins and promotes neurodegeneration.^60^*MAPT* is central to the pathogenesis of multiple neurodegenerative disorders, including Alzheimer’s disease, Parkinson’s disease and some neuropsychiatric conditions.^32^^,^^51^^,^^61–64^ Although it is tempting to establish direct connections between COVID-19 and neurodegeneration through *MAPT*, it is important to consider that these links could also be indirect. One possible indirect association is the previously documented involvement of *MAPT* in the phenotypes of aging-promoting interstitial lung disease^65^ and overall lung function.^66^ Therefore, further exploration is warranted to fully understand the relationship between COVID-19 and neurodegeneration, taking into account potential indirect pathways involving *MAPT*.

The study of *WNT3* involvement in the intersection of COVID-19 and cognitive phenotypes closely follows that of *MAPT*. While *WNT3* is involved in intelligence and multiple psychiatric conditions,^51^^,^^67^^,^^68^ its roles in COVID-19 phenotypes are more elusive and likely defined by indirect relationships with blood–brain barrier permeability.^69–71^

Colocated genes are rarely separated by recombination and are commonly coregulated. When analysis of coregulation was performed for *MAPT*-associated gene units, the levels of transcripts produced by *LRRC37A2*, *KANSL1*, *ARL17B*, *LRRC37A* and *ARL17A* were found to be affected by the *MAPT* haplotype in a dose-dependent manner.^72^ Although each of these genes may have a distinct impact on COVID-19, neurodegenerative phenotypes or both, the existence of embedded coregulation adds complexity to the study of this region. Therefore, it is crucial to conduct functional investigations both *in vitro* and in model animals to gain a deeper understanding of the interplay between these genes and their roles in the context of COVID-19 and neurodegeneration.

The composed map of the functional pathways (Figure 3B) revealed that COVID-19 influences the structure and function of multiple brain regions, including the hippocampal gyrus, orbitofrontal cortex and olfactory cortex.^24^^,^^73^^,^^74^ In both survivors of severe COVID-19 and elderly individuals, the loss of brain tissue may lead to cognitive decline.^23^^,^^75^ The correlations between changes in brain structure and age-related cognitive decline have been extensively documented in the latter group. For instance, among elderly individuals, a notable reduction in the mean volume of the right parahippocampal gyrus corresponds to their cognitive decline.^76^ The changes in the frontal cortex, especially the orbitofrontal cortex, cingulate cortex and amygdala, are associated with emotional and cognitive impairments.^77^ The subjective cognitive decline in patients is also connected to significantly reduced activation in the bilateral primary olfactory cortex.^78^ Moreover, COVID-19 may also lead to dysfunctions in the immune system, the peripheral nervous system and the lining of microvessels,^19^^,^^21^^,^^79^ a set of pathological features commonly associated with cognitive decline.^80–82^ Taken together, the functional pathways presented in Figure 3 may provide some insights into the causal effect of COVID-19 on intellectual impairment.

A limitation of this study is that the sample datasets were derived solely from European populations. To validate the findings, it is necessary to incorporate additional datasets from various population regions.

## Conclusions

In summary, our study suggests that COVID-19 may contribute to cognitive impairment. Functional variation within the tau locus and the genes of the Wnt signaling pathway may be relevant to COVID-19 and especially to its neurological sequelae.

## Supplementary Material

hcad122_Supplementary_Data
