# Supplementary material for: Causal associations and genetic overlap between COVID-19 and intelligence
Source: QJM. 2023 Jun 7;116(9):766–73. doi: 10.1093/qjmed/hcad122 (PMC10559337; doi:10.1093/qjmed/hcad122)

**Supplementary File 1. Subnetwork enrichment analysis of the ten genes overlapped between COVID-19 and intelligence.**


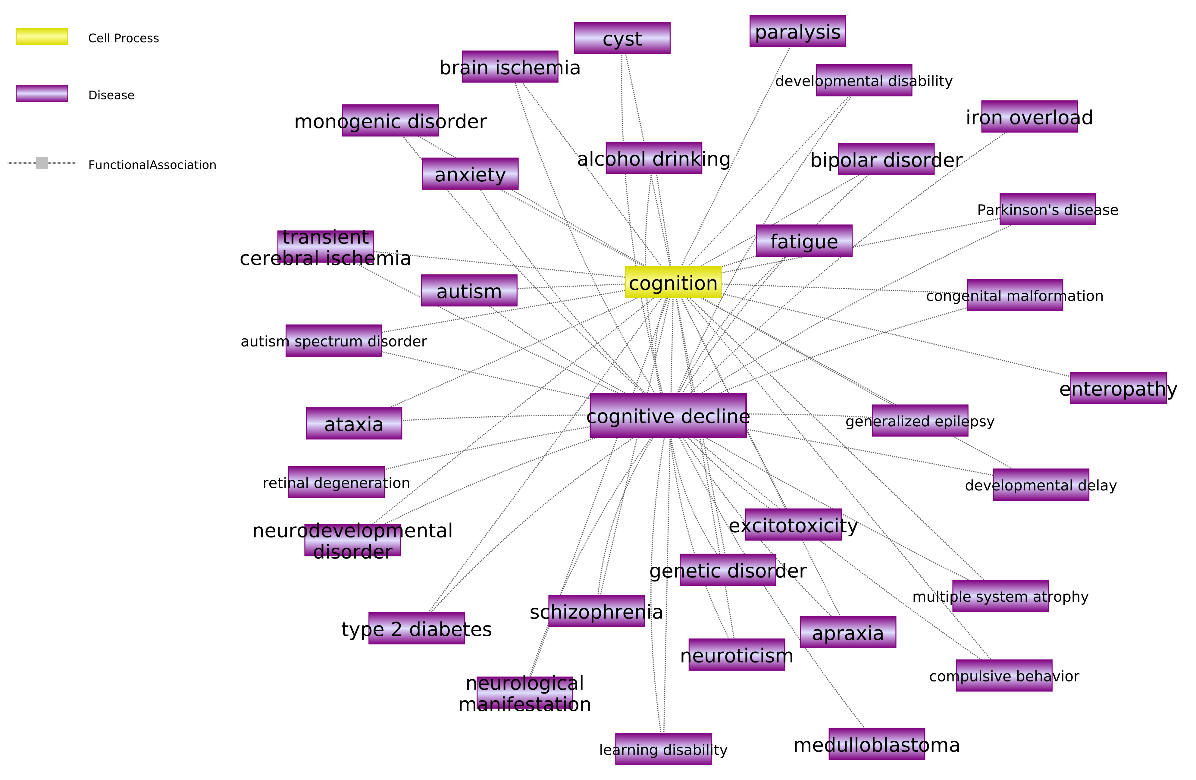

Supplement: hcad122_Supplementary_Data [file hcad122_supplementary_data.zip › hcad122_Supplementary_Data/Supplementary Figure 1 Enriched diseases_cognition.docx]
